# Nanopore sequencing for detecting reciprocal translocation carrier status in preimplantation genetic testing

**DOI:** 10.1186/s12864-022-09103-5

**Published:** 2023-01-02

**Authors:** Qiuping Xia, Shenglan Li, Taoli Ding, Zhen Liu, Jiaqi Liu, Yanping Li, Huimin Zhu, Zhongyuan Yao

**Affiliations:** 1 Reproductive Medicine Center, Xiangya Hospital, Central South University, 410008 Changsha, Hunan China; 2 Department of Gastroenterology, Xiangya Hospital, Central South University, 410008 Changsha, Hunan China; 3 Yikon Genomics Co., Ltd, 215000 Suzhou, Jiangsu China; 4 Center for Medical Genetics and Hunan Key Laboratory of Medical Genetics, School of Life Sciences, Central South University, 410008 Changsha, Hunan China

**Keywords:** Nanopore sequencing, Reciprocal translocation, Breakpoints, MaReCs, PGT-SR, Preimplantation genetic testing

## Abstract

**Background:**

Balanced reciprocal translocation (BRT) is one of the most common chromosomal abnormalities that causes infertility, recurrent miscarriage, and birth defects. Preimplantation genetic testing (PGT) is widely used to select euploid embryos for BRT carriers to increase the chance of a healthy live birth. Several strategies can be used to distinguish reciprocal translocation carrier embryos from those with a normal karyotype; however, these techniques are time-consuming and difficult to implement in clinical laboratories. In this study, nanopore sequencing was performed in two reciprocal translocation carriers, and the results were validated using the next-generation sequencing-based method named, “Mapping Allele with Resolved Carrier Status” (MaReCs).

**Results:**

The translocation breakpoints in both reciprocal translocation carriers were accurately identified by nanopore sequencing and were in accordance with the results obtained using MaReCs. More than one euploid non-balanced translocation carrier embryo was identified in both patients. Amniocentesis results revealed normal karyotypes, consistent with the findings by MaReCs and nanopore sequencing.

**Conclusion:**

Our results suggest that nanopore sequencing is a powerful strategy for accurately distinguishing non-translocation embryos from translocation carrier embryos and precisely localizing translocation breakpoints, which is essential for PGT and aids in reducing the propagation of reciprocal translocation in the population.

**Supplementary Information:**

The online version contains supplementary material available at 10.1186/s12864-022-09103-5.

## Background

Balanced reciprocal translocation (BRT) is a common chromosomal structural rearrangement (SR) caused by the interchange of two terminal segments between non-homologous chromosomes with an estimated incidence of 0.2% [1, 2]. BRT carriers are generally phenotypically normal because the overall chromosome complement remains unchanged, and the vast majority of breakpoints occur in nonrepetitive regions [2–4]. However, BRTs have been suggested to be associated with various clinical diseases in approximately 5% of cases [5, 6]. BRT carriers are at an increased risk of infertility, recurrent miscarriage, or delivery of abnormal offspring due to a poor chance of producing normal or balanced gametes as compared to normal subjects [7–9].

Preimplantation genetic testing (PGT) has been extensively used to identify normal or balanced diploid embryos in BRT carriers. Importantly, carriers can avoid physical and psychological trauma due to recurrent miscarriage or termination of the affected pregnancy upon selective transfer of normal or balanced diploid embryos. Several methods have been applied to PGT-SR, including fluorescence in situ hybridization (FISH) [10, 11], single-nucleotide polymorphism (SNP) arrays [12, 13], array comparative genomic hybridization (aCGH) [8, 14], and next-generation sequencing (NGS) [15–17]. The initial FISH method is limited to the detection of several specific chromosomes, and the results are sometimes uncertain due to ambiguous optical signals of the fluorescent probes and complex sample preparation procedures [17–20]. In recent years, aCGH and NGS have been used to screen all 24 chromosomes. In particular, SNP arrays offer a way to detect polyploidy and uniparental disomy [21, 22]. However, these methods cannot distinguish between euploid carriers and non-carrier embryos. Although transferring translocation-carrying balanced embryos should result in phenotypically normal live births, the offspring will encounter the same associated pregnancy risks as their parents when they reach reproductive age. It has recently been demonstrated that a few strategies, such as a combination of mate pair sequencing and PCR breakpoint analysis, SNP array-based comprehensive chromosome screening (CCS), NGS following microdissecting junction region (MicroSeq), and an NGS-based method named “Mapping Allele with Resolved Carrier Status” (MaReCs) can distinguish between normal and carrier embryos [16, 17, 23, 24]. However, these methods have limitations, such as a time-consuming and complex procedure for sample preparation, requirement for highly specialized equipment, availability of a reference embryo to construct allelic haplotypes using available SNPs, and difficulties in data analysis [16, 17].

Over the past few years, third-generation sequencing (TGS) has opened a new era of DNA sequencing and is an effective strategy in chromosome analysis, especially in structural chromosome rearrangements [9, 25–28]. Oxford Nano and PacBio are two common instruments used for TGS. Oxford Nanopore sequencing provides a direct and long read length, with simple library preparation, minimal capital cost, and less user time, on a small handheld platform [9, 29]. In the present study, we aimed to analyze the concordance between the results of nanopore sequencing and MaReCs, which were further confirmed by carrying out amniocentesis, to validate the feasibility of nanopore sequencing in distinguishing normal embryos from carrier embryos in PGT-SR cycles.

## Results

### MaReCs identifies translocation breakpoints

**Fig. 1:**
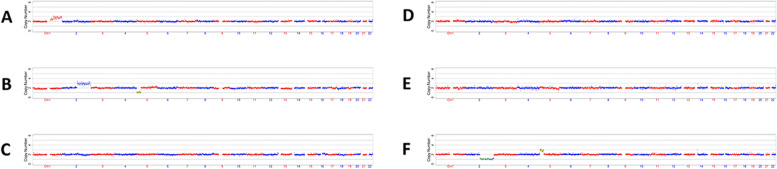
Chromosomal ploidy results of the embryos (**A**–**F**) from patient 1. In this pedigree, a total of 11 embryos (A–K) were obtained and subjected to chromosomal analysis, and the chromosomal ploidy results of the embryos (G–K) were supplied in Supplementary Figure S1 in Additional file 1. Three embryos, C, D, and E, had normal ploidy. Embryos B and F, were identified as having abnormal chromosomes 2 and 5 and were used as reference embryos for the identification of translocation breakpoint. Abnormal chromosome 1 and trisomy 15p mosaicism was detected in embryo A. Red and blue dots indicate the copy number of different chromosomes. Each point is at 1 Mb resolution. The green horizontal line indicates an abnormal copy number

**Fig. 2:**
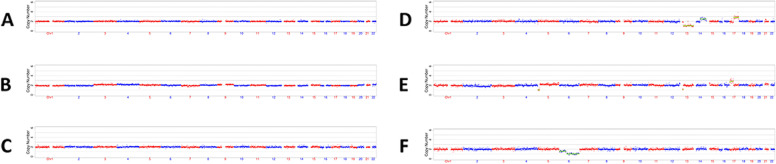
Chromosomal ploidy results of the embryos (**A**–**F**) from patient 2. Twelve embryos, A–L, were subjected to chromosomal analysis, and the chromosomal ploidy results of the embryos (G–L) were supplied in Supplementary Figure S2 in Additional file 1. Three embryos, A, B, and C, exhibited normal ploidy. Two reference embryos (**D** and **E**) with abnormal chromosomes 13 and 17 were successfully identified. Embryo F with monosomy 6q was also observed. Red and blue dots indicate the copy number of different chromosomes. Each point is at 1 Mb resolution. The green horizontal line indicates an abnormal copy number

**Fig. 3:**
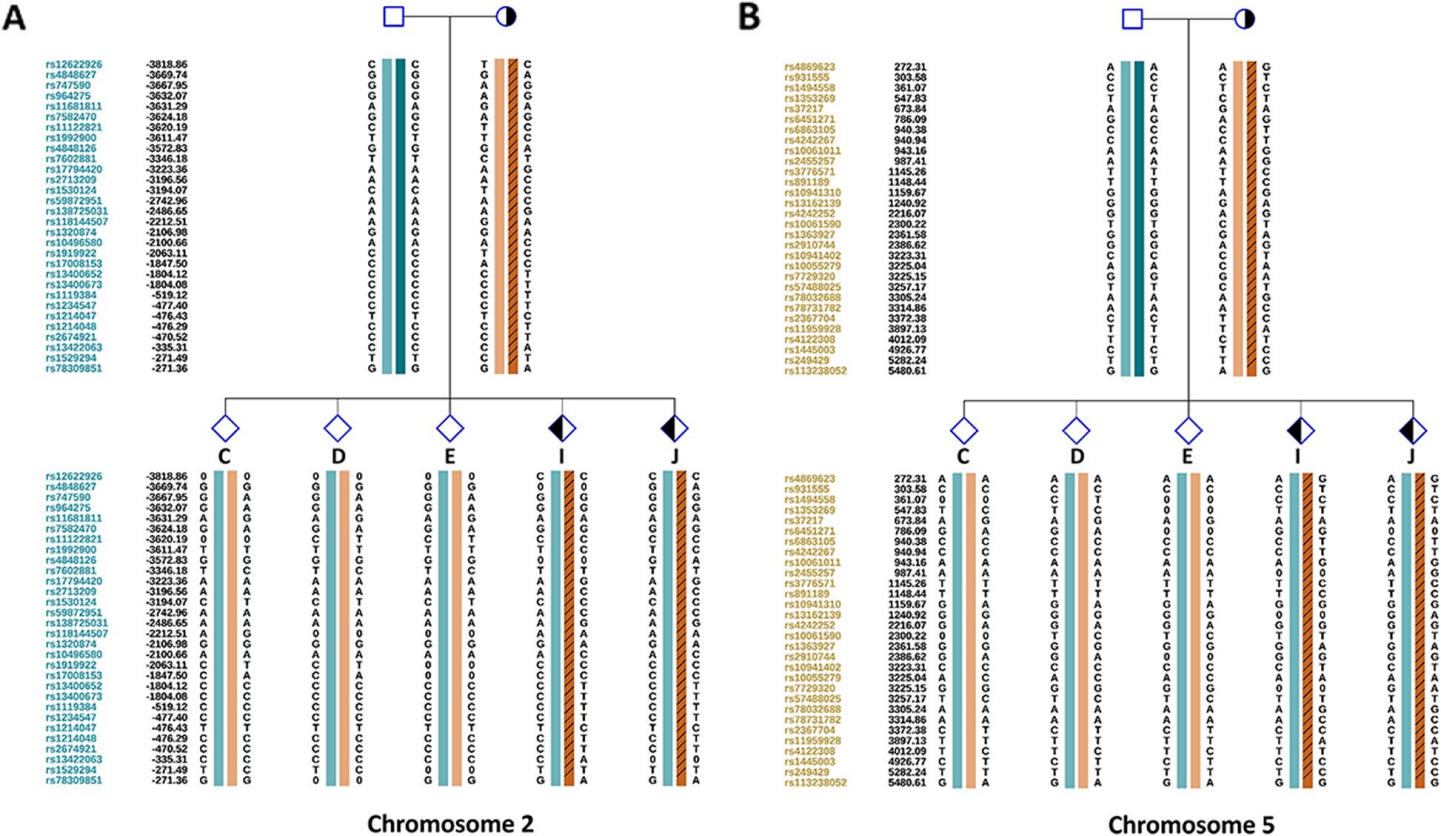
Allelic haplotype mapping to identify the carrier status of the embryos from patient 1. The two breakpoints were identified by analysis of copy number variations from the reference embryos. Subsequently, NGS was used to sequence SNPs flanking the breakpoint for the related embryos and parents. **A** and **B** SNP haplotypes were determined successfully for chromosomes 2 and 5, and three balanced diploid embryos (C, D, and E) were free of translocation

**Fig. 4:**
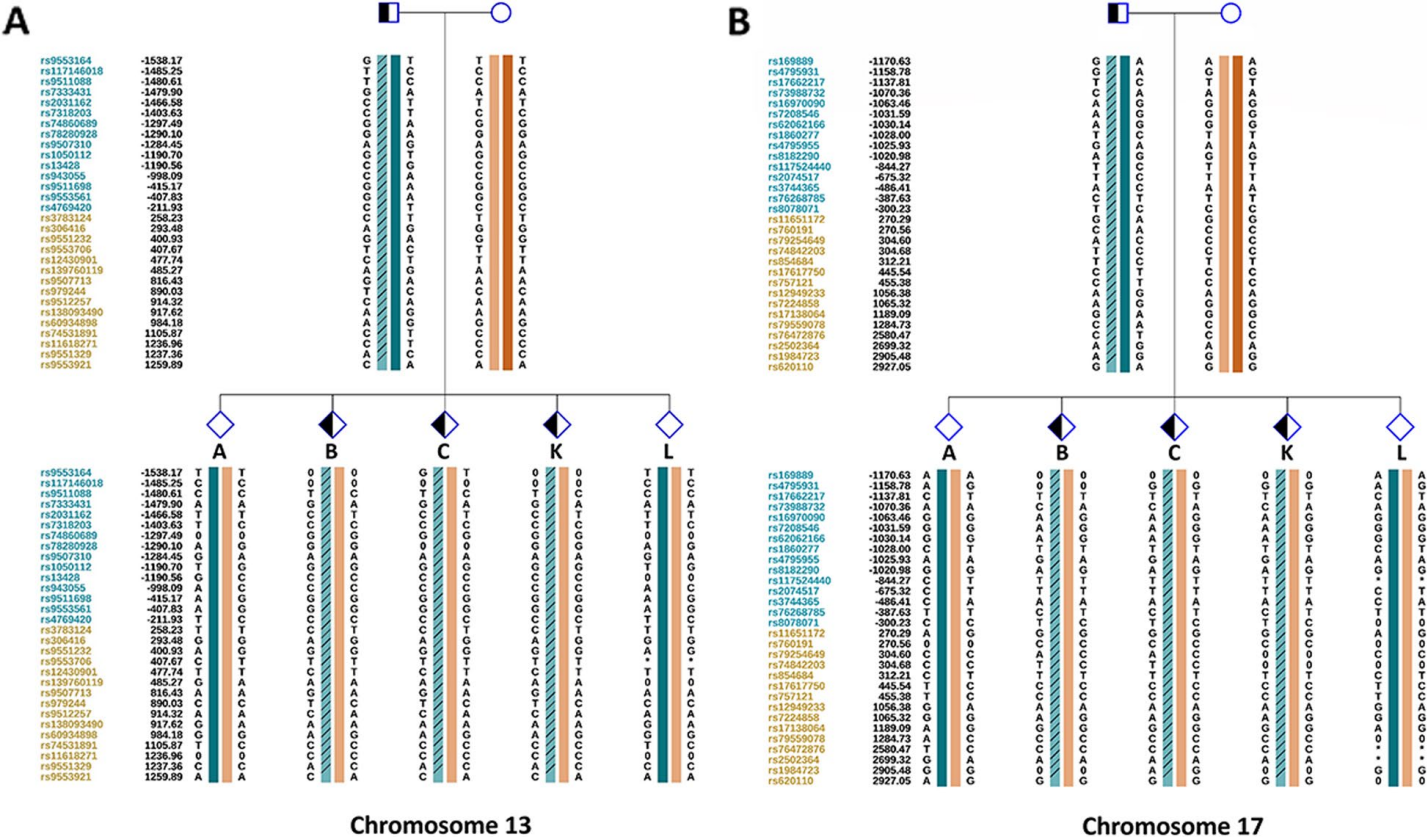
Allelic haplotype mapping to identify the carrier status of the embryos from patient 2. The two breakpoints were identified by analysis of copy number variations from the reference embryos. Subsequently, NGS was used to sequence SNPs flanking the breakpoint for the related embryos and parents. **A** and **B** SNP haplotypes were determined successfully for chromosomes 13 and 17, and two balanced non-translocation carrier embryos (A and L) were identified

We first used MaReCs to distinguish embryos with a normal karyotype from translocation-carrier embryos with balanced chromosomal ploidy. Embryos with more than one unbalanced chromosome inherited from the parent carrying the translocation were regarded as reference embryos. The chromosomal ploidy results of the embryos from the patients are shown in Figs. 1 and 2 (for additional images, see Supplementary Figures S1 and 2 in Additional file 1). In patient 1, 11 embryos (A–K) were subjected to chromosomal analysis. Briefly, embryos C, D, E, and J exhibited normal ploidy (Fig. 1; Additional file 1, Supplementary Figure S1). In contrast, embryos B, F, G, H, and K had abnormal chromosomes 2 and 5 and were used as reference embryos for the identification of translocation breakpoints (Fig. 1; Additional file 1, Supplementary Figure S1; Table 1). Abnormal chromosome 1 and trisomy 15p mosaicism were detected in embryos A and I (Fig. 1; Additional file 1, Supplementary Figure S1). Twelve embryos from patient 2 were enrolled in this study. Normal ploidy was confirmed in four embryos: A, B, C, and L (Fig. 2; Additional file 1, Supplementary Figure S2). Five reference embryos (D, E, G, H, and I) with abnormal chromosomes 13 and 17 were successfully identified (Fig. 2; Additional file 1, Supplementary Figure S2; Table 1). Two embryos (F and J) with other abnormal chromosomes were also observed, and monosomy 10q mosaicism was detected in embryo K (Fig. 2; Additional file 1, Supplementary Figure S2). To further characterize the translocation breakpoints, copy number variations from the reference embryos in both pedigrees were analyzed. All four breakpoints were identified (Table 2). Subsequently, we used NGS to sequence SNPs flanking the breakpoint in the related embryos and parents. SNP haplotypes were conducted for both pedigrees, which enabled the mapping of the haplotypes linked to the translocation-carrying chromosome and the normal chromosome (Figs. 3 and 4). Three euploid embryos from patient 1 were free of translocation (Fig. 3), and two similar embryos were identified in patient 2 (Fig. 4). Taken together, we successfully identified breakpoints for both pedigrees using MaReCs, which enabled the transfer of a balanced translocation-free embryo to the patients.

**Table 1:** Summary of chromosome ploidy results of the reference embryos by MaReCs

**Patient**	**Embryo ID**	**Chromosome ploidy result**	**Reference embryo**
Patient 1	B	46, XN, +2q(q14.3→q37.3,~118Mb,×3), -5p(pter→p13.2,~35Mb,×1)	Yes
	F	46, XN, -2q(q14.3→q14.3,~5Mb,×1), -2q(q21.1→q37.1,~104Mb,×1), -2q(q37.1→q37.3,~8Mb,×1), +5p(pter→p13.2,~35Mb,×3)	Yes
	G	46, XN, -2q(q14.3→q37.3,~118Mb,×1), +5p(p15.33→p13.2,~34Mb,×3), +6q(q11.1→q14.1,~20Mb,×3,mos,~31%), -10q(q24.2→q25.1,~11Mb,×1,mos,~33%)	Yes
	H	46, XN, +2q(q14.3→q37.3,~118Mb,×3), -5p(pter→p13.2,~35Mb,×1)	Yes
	K	46, XX, +Xp(p21.3→p11.4,~14Mb,×3,mos,~31%), -2q(q14.3→q37.3,~118Mb,×1), +5p(pter→p13.2,~35Mb,×3)	Yes
Patient 2	D	46, XN, -13q(q12.13→q34,~88Mb,×1), +14q(q22.1→q32.33,~52Mb,×3,mos,~51%), +17q(q12→q25.3,~46Mb,×3)	Yes
	E	46, XN, -5p(pter→p15.1,~16Mb,×1), -13q(q12.11→q12.12,~6Mb,×1), +17p(×3), +17q(q11.2→q12,~8Mb,×3)	Yes
	G	46, XN, +13q(q12.13→q34,~88Mb,×3), -17q(q12→q25.3,~46Mb,×1)	Yes
	H	46, XN, -13q(q12.13→q34,~88Mb,×1), +17q(q12→q25.3,~46Mb,×3)	Yes
	I	46, XN, +13q(q12.13→q34,~88Mb,×3), -17q(q12→q25.3,~46Mb,×1)	Yes

**Table 2:** Translocation breakpoint characteristics identified by MaReCs

Patient	Cytogenetic results	Number of embryos tested	Breakpoint location
Patient 1	46,XX,t(2;5)(q14.2;p13.1)	11	chr2: 125,200,001 ± 200k; chr5: 35,500,001 ± 200k
Patient 2	46,XY,t(13;17)(q11;q11.2)	12	chr13: 26,200,001 ± 200k; chr17: 34,000,001 ± 200k

### Nanopore sequencing-based mapping of the translocation breakpoints

**Fig. 5:**
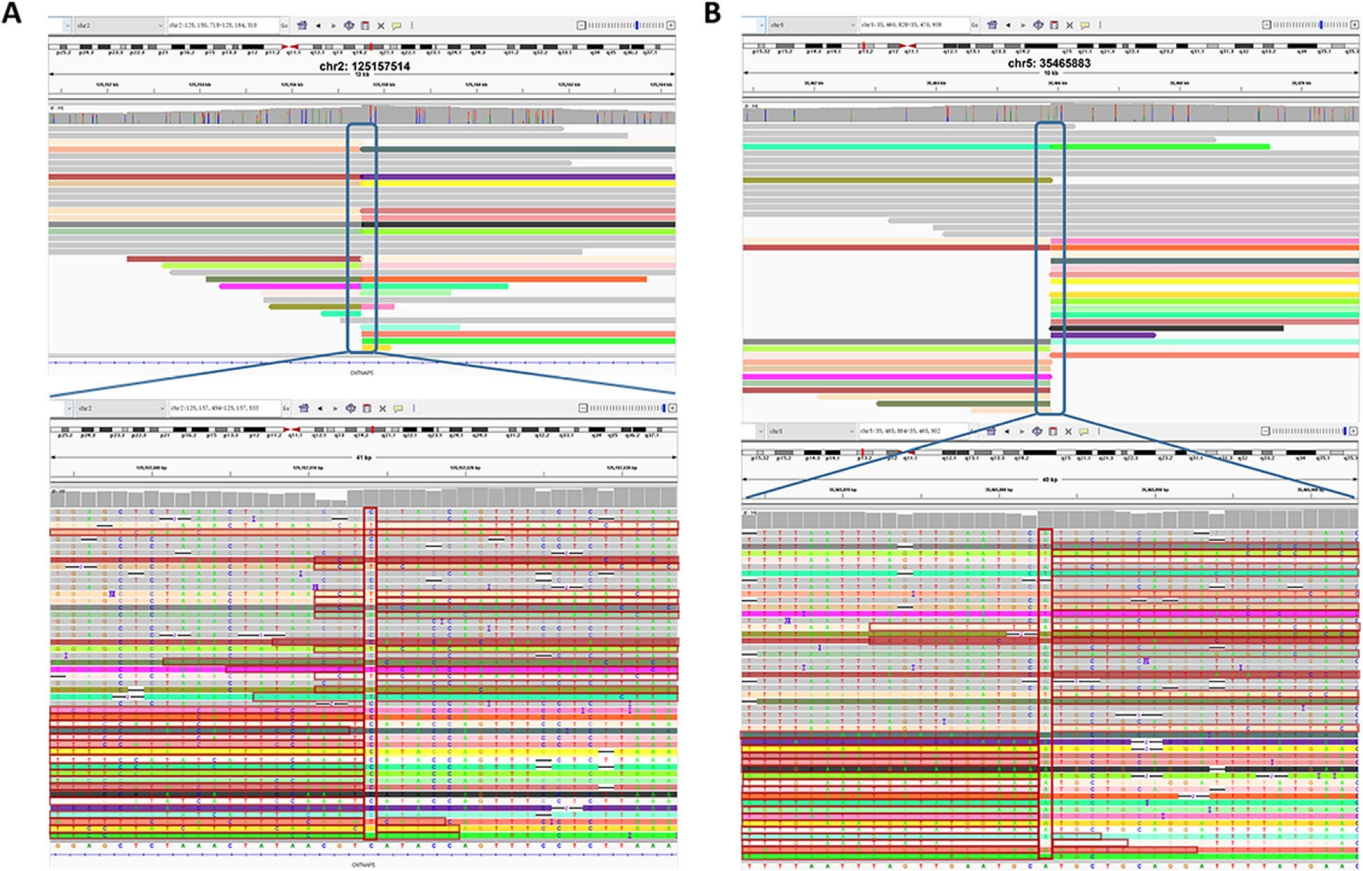
Nanopore sequencing mapping of translocation breakpoints in patient 1. Nanopore sequencing was performed on a PromethION 48. **A** and **B** Breakpoints identified by nanopore sequencing were located at chr2:125,157,514 and chr5:35,465,883

**Fig. 6:**
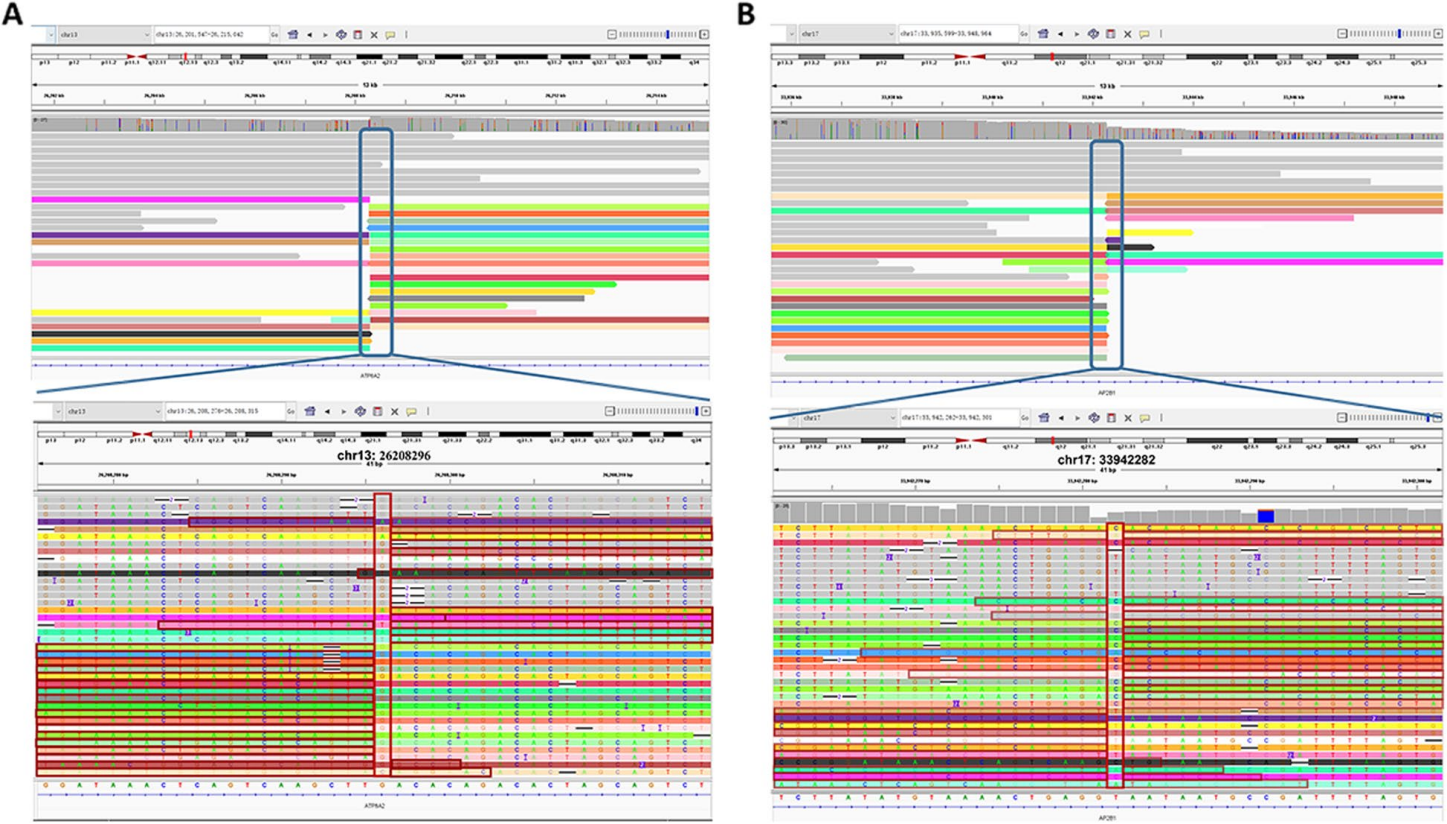
Nanopore sequencing mapping of translocation breakpoints in patient 2. Nanopore sequencing was performed on a PromethION 48. **A** and **B** Breakpoints identified by nanopore sequencing were located at chr13:26,208,296 and chr17:33,942,282

**Fig. 7:**
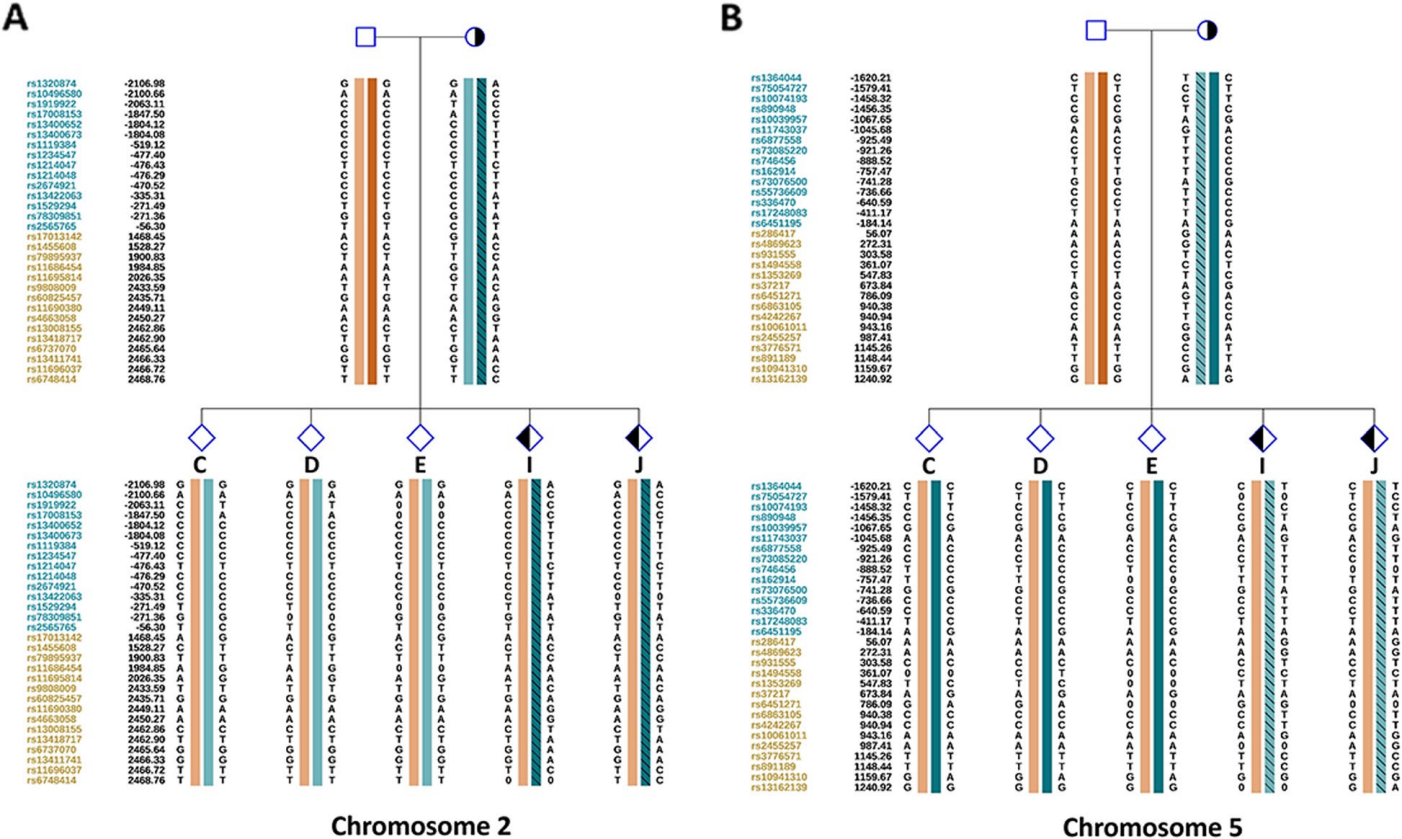
Resolving the carrier status of the embryos from patient 1 by nanopore sequencing mapping. **A** and **B** SNP haplotypes were determined successfully for chromosomes 2 and 5, and three balanced diploid embryos (C, D, and E) were free of translocation. Detailed SNP haplotypes were supplied in Supplementary Figures S3 and 4 in Additional files 4 and 5

**Fig. 8:**
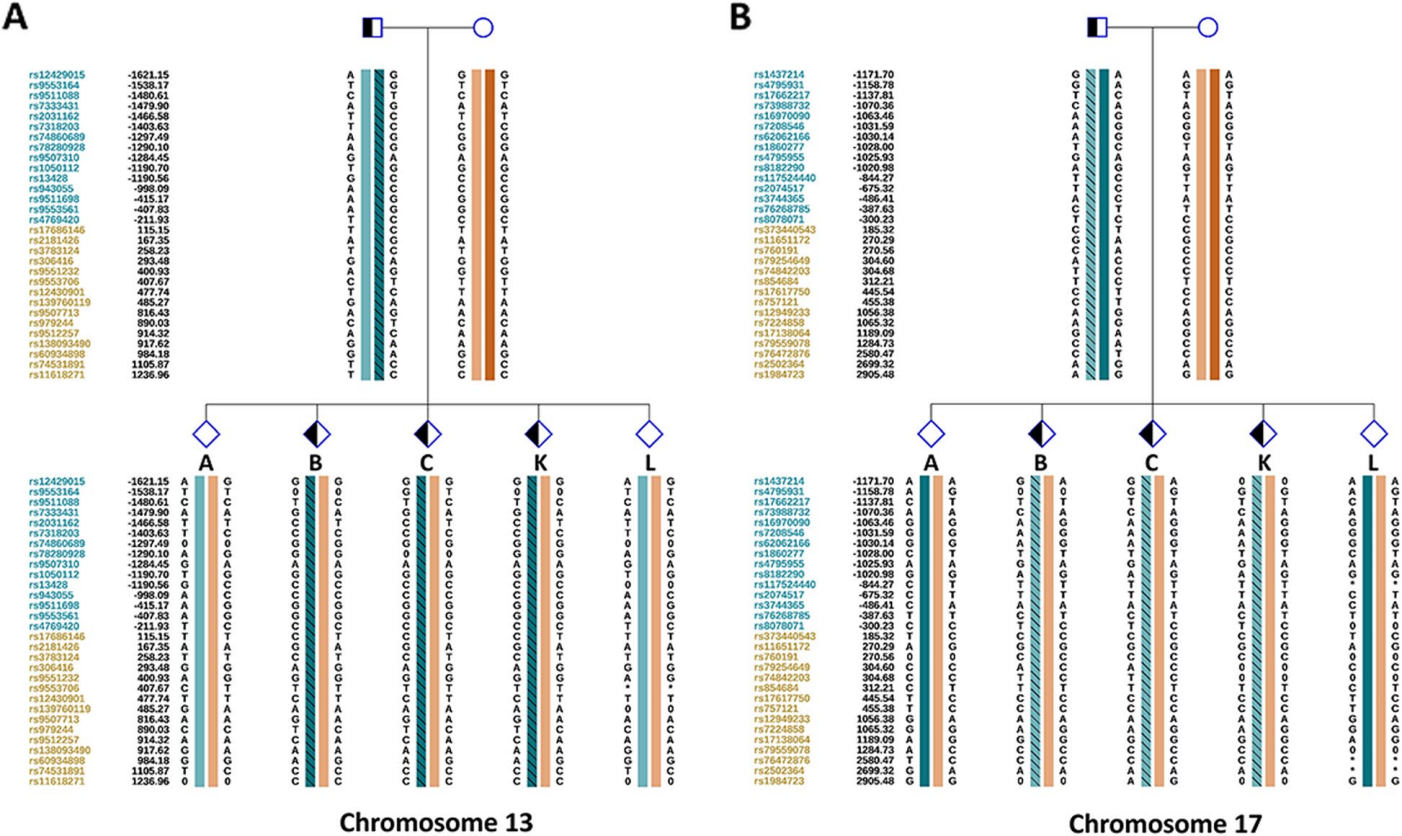
Resolving the carrier status of the embryos from patient 2 by nanopore sequencing mapping. **A** and **B** SNP haplotypes were determined successfully for chromosomes 13 and 17, and two balanced non-translocation carrier embryos (A and L) were identified. Detailed SNP haplotypes were supplied in Supplementary Figures S5 and 6 in Additional files 6 and 7

TGS was performed on a PromethION 48 (Oxford Nanopore Technologies, Oxford, UK) with a single sample loaded on one flow cell. We obtained 101 Gb of data for patient 1 and 103 Gb of data for patient 2 (Additional files 2 and 3). The mean read length for patient 1 was 19,796 bp, and N50 was 26,324 bp, with an average depth of ~ 28.89× (Additional file 2). The mean read length for patient 2 was 17,361 bp, and N50 was 23,138 bp, with an average depth of ~ 29.4× (Additional file 3). We analyzed the nanopore sequencing data obtained from PromethION 48 and successfully identified the translocation breakpoints in both patients. Breakpoints were located at chr2:125,157,514 and chr5:35,465,883 in patient 1 (Fig. 5A, B) and chr13:26,208,296 and chr17:33,942,282 in patient 2 (Fig. 6A, B), which was consistent with the MaReCs results. Additionally, the SNP haplotypes constructed using nanopore sequencing data were consistent with those obtained by MaReCs (compare Fig. 7 with Figs. 3 and 8 with Fig. 4). These findings indicate that nanopore sequencing accurately identifies translocation breakpoints, which can be used to distinguish translocation-free embryos from balanced diploid embryos in PGT-SR cycles.

### Prenatal follow-up

Embryos D and A were frozen day-5 blastocysts with Gardner’s grades 5CB and 4BB, respectively, that were transferred into the uteruses of patients 1 and 2, respectively, with complete informed consent, resulting in two singleton pregnancies. Amniocentesis was performed in both patients at 18–20 weeks of gestation. The results revealed normal karyotypes, consistent with the findings of MaReCs and nanopore sequencing (data not shown). Both pregnancies are currently ongoing. Postnatal follow-up needs to be performed to confirm consistency among the results of the PGT-SR cycle, amniocentesis, and peripheral blood analysis.

## Discussion

In this study, nanopore sequencing was used to distinguish non-carrier embryos from balanced diploid embryos in PGT-SR cycles. The results were consistent with those obtained by MaReCs and were further confirmed by the karyotyping of amniocytes. Translocation breakpoints were successfully identified by nanopore sequencing with an accuracy of breakpoint determination to a single base pair. These findings strongly suggest that nanopore sequencing is one of the most appropriate clinical strategies for resolving the carrier status of balanced diploid embryos for BRT carriers.

PGT-SR has been successfully utilized in selecting balanced diploid embryos as BRT carriers, but its application in resolving the translocation carrier status of euploid embryos remains challenging. To achieve this, recent studies have established strategies such as MicroSeq-PGD and MaReCs in PGT-SR cycles [16, 17]. The MicroSeq-PGD method combines the chromosome microdissection technique with NGS followed by PGT to characterize the translocation breakpoints and flanking SNP haplotypes, which enables the resolution of non-carrier embryos from BRT carriers. However, this method fails to detect translocation breakpoints in repeated or GC-bias regions. Moreover, it requires highly specialized equipment and advanced experimental skills. These limitations make it difficult to widely apply the PGT-SR cycle. MaReCs reportedly enables chromosomal ploidy screening and the resolution of the translocation carrier status of balanced diploid embryos. However, this method relies on the determination of informative SNPs flanking the translocation breakpoints, which is possible only when a reference embryo is available. Another limitation of MaReCs is that translocation-free embryos cannot be identified using the translocation breakpoints located in repeated regions.

Nanopore sequencing is a single-molecule long-read sequencing technology with several advantages. First, it provides long read lengths, which greatly increases the chance of precisely identifying translocation breakpoints. Second, it can address more complex translocations and is especially helpful in resolving breakpoints located in repetitive and GC-bias regions [30]. Third, it is useful for mapping SNP haplotypes linked to translocation, which is critical for the identification of non-carrier embryos in the PGT-SR cycle. Furthermore, compared to other available methods, nanopore sequencing requires less time and cost, which is of particular importance for patients undergoing PGT cycles. More importantly, translocation breakpoints can be identified in genomic DNA extracted from the peripheral blood of BRT carriers using nanopore sequencing prior to the initiation of the PGT-SR cycle. These advantages will allow for nanopore sequencing to be used for preventing recurrent miscarriages in PGT cycles once it is available in most PGT centers in the future.

Translocation breakpoint identification is a challenge in the PGT-SR cycle, and several methods have been established to map breakpoints to kilobase levels. However, these methods are usually time-consuming and difficult to widely use in the population. In this study, we observed that MaReCs identified translocation breakpoints at the kilobase level (approximately 200 kb). In contrast, we mapped translocation breakpoints to a higher resolution, that is, at the single-base level, using nanopore sequencing. Taken together, these observations indicate that long reads (nanopore sequencing) are superior to short reads (NGS) for the detection of translocation breakpoints; this finding shows broad prospects for clinical applications in blocking translocation propagation in the population. Nonetheless, we always suggest to the patients that they should not discard any balanced translocation carrier embryos when they have several euploid non-translocation carrier embryos. We also inform the patients that the balanced translocation carrier embryos can be thawed and transferred. Indeed, transferring a balanced translocation carrier embryo should result in phenotypically normal live birth.

However, nanopore sequencing has certain limitations. One such limitation is that it is not possible to identify the translocation breakpoints of Robertsonian translocation carriers or those located in the gap regions of the human genome. In addition, high-molecular-weight genomic DNA is essential for nanopore sequencing to yield long reads, but most PGT centers employ methods that tend to be more well-suited for short-read sequencing. Furthermore, since PGT centers usually tend to use conventional spin-column for genomic DNA extractions, it can be difficult to achieve a total amount of 2 µg DNA. Obtaining large amounts of peripheral blood samples that are required to achieve the desired amount of genomic DNA can also be a challenge for PGT centers. Finally, the cost of short-read sequencing has been decreasing, which will allow for NGS to be used for PGT-SR on patients at a reasonable cost, especially since the cost of the entire nanopore sequencing workflow is also a critical point in the PGT centers in daily practice.

## Conclusion

In conclusion, we demonstrated the feasibility of nanopore sequencing to accurately detect translocation breakpoints in BRT carriers and that nanopore sequencing can be used to distinguish translocation-free embryos from balanced diploid embryos in clinical PGT-SR cycles. This study provides important information for the improvement of genetic counseling guidelines regarding BRT and will eventually help BRT carriers. It is imperative to conduct large-scale studies to validate the specificity and sensitivity of nanopore sequencing in the future.

## Methods

### Patients

Two balanced reciprocal translocation carriers who underwent PGT-SR cycles in the Reproductive Medicine Center, Xiangya Hospital, Central South University were enrolled in this study. Patient 1 experienced one miscarriage followed by a 5-year history of secondary infertility. Patient 2 experienced two ectopic pregnancies, and asthenozoospermia was confirmed in the male patient. The karyotypes were 46,XX,t(2;5)(q14.2;p13.1) and 46,XY,t(13;17)(q11;q11.2). Complete informed consent was obtained from the couples. Blood samples were collected from the couples.

### In vitro fertilization, blastocyst biopsy, and whole genome amplification (WGA)

Metaphase II oocytes were fertilized via intracytoplasmic single-sperm injection following a standard protocol and then cultured for 5–6 days to develop into the blastocyst stage. Three to five trophectoderm cells were biopsied from fresh day-5/6 blastocysts and placed into PCR tubes, which were then subjected to WGA by multiple annealing and looping-based amplification cycles (MALBAC). IVF, embryo culture, blastocyst biopsy, and cryopreservation procedures were performed at Reproductive Medicine Center, Xiangya Hospital, Central South University, using previously published procedures. In total, 23 embryos from two pedigrees were obtained and detected.

### DNA extraction

Peripheral blood samples were collected, and high-molecular-weight genomic DNA was prepared using the SDS method followed by purification with the QIAGEN® Genomic kit (Cat#13,343, QIAGEN, Germany) according to the standard operating procedure provided by the manufacturer [31]. DNA degradation and contamination were monitored using 1% agarose gels. DNA purity was detected using a NanoDrop™ One UV-Vis spectrophotometer (Thermo Fisher Scientific, USA): OD260/280 ranged from 1.8 to 2.0 and OD 260/230 was 2.0–2.2. Finally, the DNA concentration was measured using a Qubit® 3.0 Fluorometer (Invitrogen, USA).

### Library preparation and nanopore sequencing

DNA (2 µg per sample) was used as the input material for Oxford Nanopore Technologies library preparation. After the sample was qualified, size selection of long DNA fragments was performed using the BluePippin system (Sage Science, USA). Next, the ends of the DNA fragments were repaired, and the A-ligation reaction was conducted using the NEBNext Ultra II End Repair/dA-tailing Kit (Cat# E7546, NEB, USA). The adapter in the LSK109 kit was used for further ligation, and a Qubit® 3.0 Fluorometer was used to quantify the size of the library fragments. Sequencing was performed using a PromethION 48 (Oxford Nanopore Technologies, UK).

### Data quality control

Nanopore sequencers output FAST5 files containing the signal data, which were first converted to FASTQ format using Guppy. The raw reads in FASTQ format with mean_qscore_template < 7 were then filtered, resulting in pass reads.

### MaReCs analysis

The detailed workflow of the MaReCs method has been described previously [17]. TE biopsy samples were subjected to MALBAC-based WGA, followed by an NGS-based CCS assay. Translocation breakpoint identification and haplotype linkage analysis were performed, which eventually enabled the selection of non-carrier embryos.

### Validation of the nanopore sequencing and MaReCs results

Pregnant patients after PGT-SR cycles were referred to the Department of Obstetrics, Xiangya Hospital Central South University, for prenatal/postnatal follow-up. Nanopore sequencing and MaReCs results were validated by karyotype analysis of amniotic fluid cells at 18–20 weeks of gestation.

## Supplementary Information

**Additional file 1: Fig.S1. **Chromosomal ploidy results of the embryos (G–K) from patient 1. Embryo J had normal ploidy. Three embryos, G, H, and K, were identified as having abnormal chromosomes 2 and 5 and were used as reference embryos for the identification of translocation breakpoint. Abnormal chromosome 1 and trisomy 15p mosaicism was detected in embryo I. Red and blue dots indicate the copy number of different chromosomes. Each point is at 1 Mb resolution. The green horizontal line indicates an abnormal copy number. **Fig.S2.** Chromosomal ploidy results of the embryos (G–L) from patient 2. Embryos L exhibited normal ploidy. Three reference embryos (G,H, and I) with abnormal chromosomes 13 and 17 were successfully identified. Embryo J with monosomy 7 was observed, and monosomy 10q mosaicism was detected in embryo K. Red and blue dots indicate the copy number of different chromosomes. Each point is at 1 Mb resolution. The green horizontal line indicates an abnormal copy number.

**Additional file 2.**

**Additional file 3.**

**Additional file 4: Fig.S3. **Informative single-nucleotide polymorphisms (SNPs) flanking the breakpoint in chromosome 2 from pedigree 1 (Family LJ) and embryos based on nanopore sequencing. SNP haplotypes were identified successfully for chromosome 2, and three balanced diploid embryos (C, D, and E) were free of translocation.

**Additional file 5: Fig.S4. **Informative single-nucleotide polymorphisms (SNPs) flanking the breakpoint in chromosome 5 from pedigree 1 (Family LJ) and embryos based on nanopore sequencing. SNP haplotypes were identified successfully for chromosome 5, and three balanced diploid embryos (C, D, and E) were free of translocation.

**Additional file 6: Fig.S5. **Informativesingle-nucleotide polymorphisms (SNPs) flanking the breakpoint in chromosome 13 from pedigree 2 (Family LHY) and embryos based on nanopore sequencing. SNP haplotypes were identified successfully for chromosome 13, and two balanced diploid embryos (A and L) were free of translocation. 

**Additional file 7: Fig.S6. **Informativesingle-nucleotide polymorphisms (SNPs) flanking the breakpoint in chromosome 17 from pedigree 2 (Family LHY) and embryos based on nanopore sequencing. SNP haplotypes were identified successfully for chromosome 17, and two balanced diploid embryos (A and L) were free of translocation.

## Data Availability

The data generated or analyzed in our study are available in this published article and its supplementary information files. The sequence data are available at https://db.cngb.org/search/?q=CNP0003353.

## Abbreviations

aCGH: Array comparative genomic hybridization
BRT: Balanced reciprocal translocation
CCS: Compressive chromosome screening
FISH: Fluorescence in situ hybridization
MaReCs: Mapping Allele with Resolved Carrier Status
MALBAC: Multiple annealing and looping-based amplification cycles
NGS: Next-generation sequencing
PGT: Preimplantation genetic testing
SNP: Single-nucleotide polymorphism
SR: Structural rearrangement
TGS: Third-generation sequencing
TE: Trophectoderm
WGA: Whole genome amplification
