# Supplementary material for: Nanopore sequencing for detecting reciprocal translocation carrier status in preimplantation genetic testing
Source: BMC Genomics. 2023 Jan 2;24:1. doi: 10.1186/s12864-022-09103-5 (PMC9809107; doi:10.1186/s12864-022-09103-5)
Supplement: Supplementary file 1 — Additional file 1: Fig.S1. Chromosomal ploidy results of the embryos (G–K) from patient 1. Embryo J had normal ploidy. Three embryos, G, H, and K, were identified as having abnormal chromosomes 2 and 5 and were used as reference embryos for the identification of translocation breakpoint. Abnormal chromosome 1 and trisomy 15p mosaicism was detected in embryo I. Red and blue dots indicate the copy number of different chromosomes. Each point is at 1 Mb resolution. The green horizontal line indicates an abnormal copy number. Fig.S2. Chromosomal ploidy results of the embryos (G–L) from patient 2. Embryos L exhibited normal ploidy. Three reference embryos (G,H, and I) with abnormal chromosomes 13 and 17 were successfully identified. Embryo J with monosomy 7 was observed, and monosomy 10q mosaicism was detected in embryo K. Red and blue dots indicate the copy number of different chromosomes. Each point is at 1 Mb resolution. The green horizontal line indicates an abnormal copy number. [file 12864_2022_9103_MOESM1_ESM.pdf]

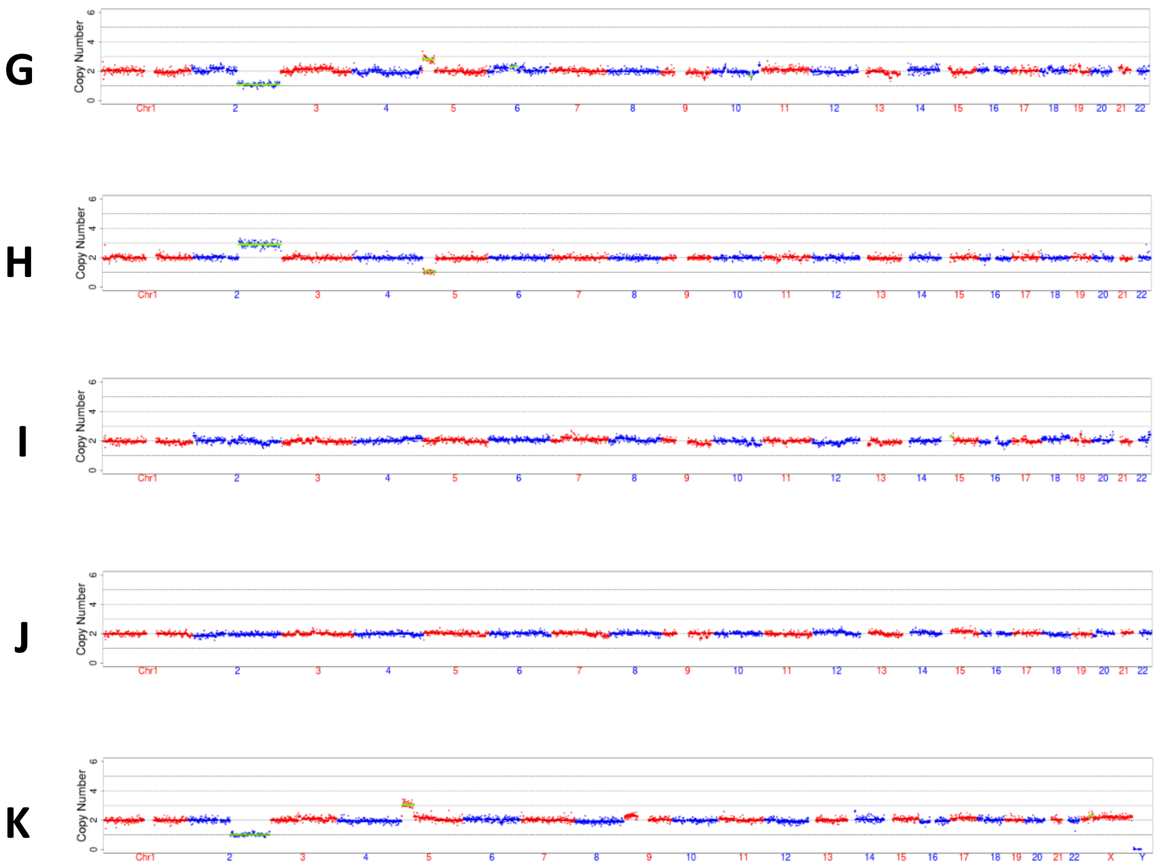

Fig. S1 Chromosomal ploidy results of the embryos (G–K) from patient 1. Embryo J had normal ploidy. Three embryos, G, H, and K, were identified as having abnormal chromosomes 2 and 5 and were used as reference embryos for the identification of translocation breakpoint. Abnormal chromosome 1 and trisomy 15p mosaicism was detected in embryo I. Red and blue dots indicate the copy number of different chromosomes. Each point is at 1 Mb resolution. The green horizontal line indicates an abnormal copy number.

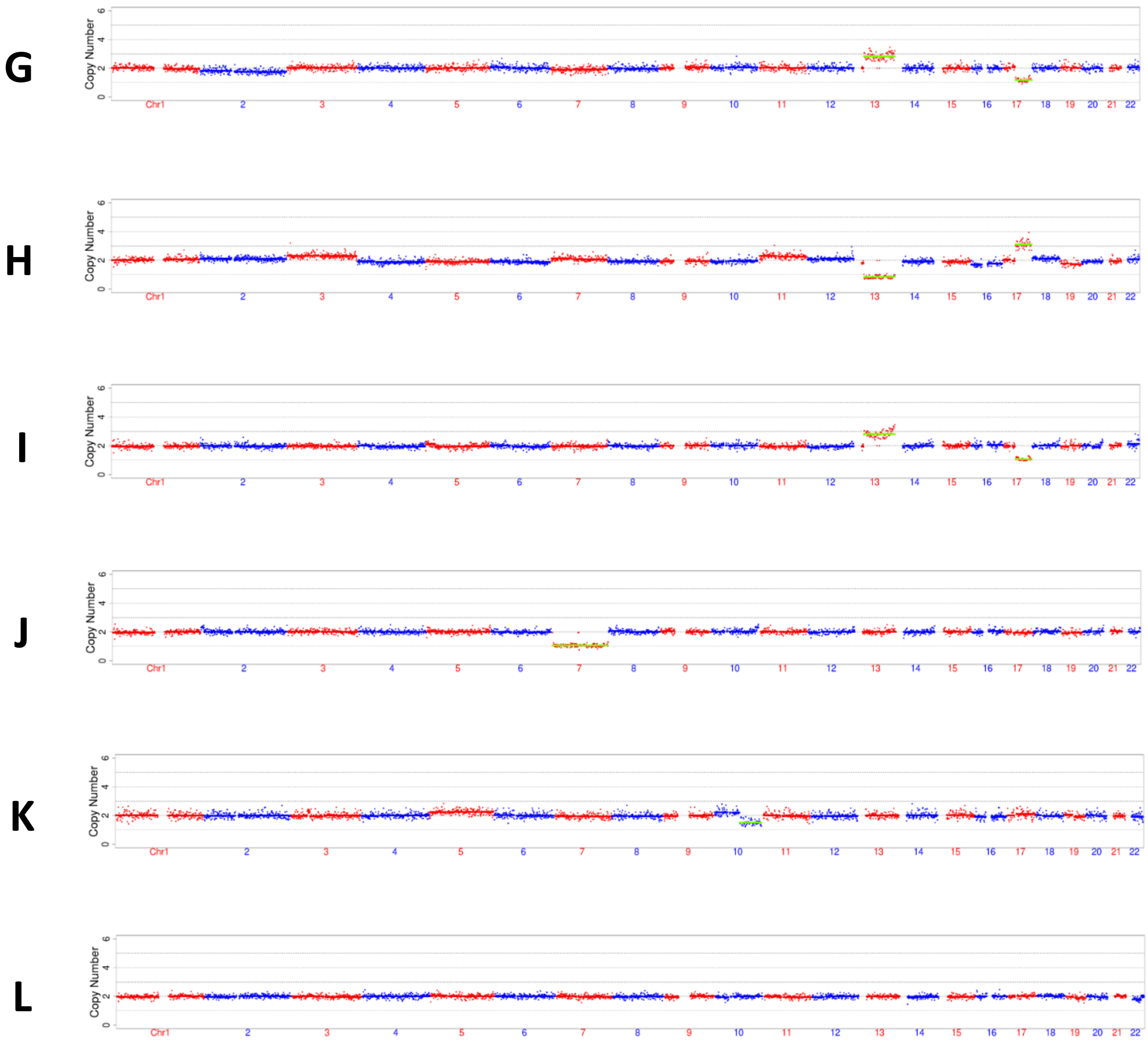

Fig. S2 Chromosomal ploidy results of the embryos (G–L) from patient 2. Embryos L exhibited normal ploidy. Three reference embryos (G, H, and I) with abnormal chromosomes 13 and 17 were successfully identified. Embryo J with monosomy 7 was observed, and monosomy 10q mosaicism was detected in embryo K. Red and blue dots indicate the copy number of different chromosomes. Each point is at 1 Mb resolution. The green horizontal line indicates an abnormal copy number.
