# Supplementary material for: Nanopore sequencing for detecting reciprocal translocation carrier status in preimplantation genetic testing
Source: BMC Genomics. 2023 Jan 2;24:1. doi: 10.1186/s12864-022-09103-5 (PMC9809107; doi:10.1186/s12864-022-09103-5)
Supplement: Supplementary file 2 — Additional file 2. [file 12864_2022_9103_MOESM2_ESM.html]

NanoPlot Report


# NanoPlot statistics report

## Menu

- Summary Statistics
- Plots
  - Weighted histogram of read lengths
  - Weighted histogram of read lengths after log transformation
  - Non weighted histogram of read lengths
  - Non weighted histogram of read lengths after log transformation
  - Yield by length
  - Read lengths vs Average read quality plot using dots
- Report issue on Github


## NanoPlot reports

### Summary statistics

|  |  |
| --- | --- |
| General summary |  |
| Mean read length | 19,796.1 |
| Mean read quality | 9.8 |
| Median read length | 17,358.0 |
| Median read quality | 10.0 |
| Number of reads | 5,112,042.0 |
| Read length N50 | 26,324.0 |
| STDEV read length | 13,513.1 |
| Total bases | 101,198,259,873.0 |
| Number, percentage and megabases of reads above quality cutoffs |  |
| >Q5 | 5112042 (100.0%) 101198.3Mb |
| >Q7 | 5112040 (100.0%) 101198.3Mb |
| >Q10 | 2576835 (50.4%) 52569.6Mb |
| >Q12 | 43074 (0.8%) 600.7Mb |
| >Q15 | 3 (0.0%) 0.0Mb |
| Top 5 highest mean basecall quality scores and their read lengths |  |
| 1 | 15.3 (232) |
| 2 | 15.2 (258) |
| 3 | 15.0 (207) |
| 4 | 15.0 (5650) |
| 5 | 14.9 (238) |
| Top 5 longest reads and their mean basecall quality score |  |
| 1 | 184691 (9.9) |
| 2 | 184107 (7.9) |
| 3 | 183701 (10.4) |
| 4 | 181567 (9.9) |
| 5 | 178870 (10.1) |

### Plots

Weighted histogram of read lengths

#### Weighted histogram of read lengths

Weighted histogram of read lengths after log transformation

#### Weighted histogram of read lengths after log transformation

Non weighted histogram of read lengths

#### Non weighted histogram of read lengths

Non weighted histogram of read lengths after log transformation

#### Non weighted histogram of read lengths after log transformation

Yield by length

#### Yield by length

Read lengths vs Average read quality plot using dots

#### Read lengths vs Average read quality plot using dots
