# Supplementary material for: Nanopore sequencing for detecting reciprocal translocation carrier status in preimplantation genetic testing
Source: BMC Genomics. 2023 Jan 2;24:1. doi: 10.1186/s12864-022-09103-5 (PMC9809107; doi:10.1186/s12864-022-09103-5)
Supplement: Supplementary file 3 — Additional file 3. [file 12864_2022_9103_MOESM3_ESM.html]

NanoPlot Report


# NanoPlot statistics report

## Menu

- Summary Statistics
- Plots
  - Weighted histogram of read lengths
  - Weighted histogram of read lengths after log transformation
  - Non weighted histogram of read lengths
  - Non weighted histogram of read lengths after log transformation
  - Yield by length
  - Read lengths vs Average read quality plot using dots
- Report issue on Github


## NanoPlot reports

### Summary statistics

|  |  |
| --- | --- |
| General summary |  |
| Mean read length | 17,361.8 |
| Mean read quality | 9.8 |
| Median read length | 16,416.0 |
| Median read quality | 10.0 |
| Number of reads | 5,928,290.0 |
| Read length N50 | 23,138.0 |
| STDEV read length | 12,206.6 |
| Total bases | 102,925,494,320.0 |
| Number, percentage and megabases of reads above quality cutoffs |  |
| >Q5 | 5928290 (100.0%) 102925.5Mb |
| >Q7 | 5928285 (100.0%) 102925.5Mb |
| >Q10 | 2888669 (48.7%) 51839.5Mb |
| >Q12 | 65695 (1.1%) 840.3Mb |
| >Q15 | 7 (0.0%) 0.0Mb |
| Top 5 highest mean basecall quality scores and their read lengths |  |
| 1 | 16.0 (234) |
| 2 | 16.0 (303) |
| 3 | 15.9 (374) |
| 4 | 15.7 (2439) |
| 5 | 15.2 (534) |
| Top 5 longest reads and their mean basecall quality score |  |
| 1 | 205419 (7.4) |
| 2 | 176712 (7.7) |
| 3 | 168948 (8.3) |
| 4 | 166435 (10.2) |
| 5 | 166274 (9.6) |

### Plots

Weighted histogram of read lengths

#### Weighted histogram of read lengths

Weighted histogram of read lengths after log transformation

#### Weighted histogram of read lengths after log transformation

Non weighted histogram of read lengths

#### Non weighted histogram of read lengths

Non weighted histogram of read lengths after log transformation

#### Non weighted histogram of read lengths after log transformation

Yield by length

#### Yield by length

Read lengths vs Average read quality plot using dots

#### Read lengths vs Average read quality plot using dots
