# Supplementary material for: Nanopore sequencing for detecting reciprocal translocation carrier status in preimplantation genetic testing
Source: BMC Genomics. 2023 Jan 2;24:1. doi: 10.1186/s12864-022-09103-5 (PMC9809107; doi:10.1186/s12864-022-09103-5)
Supplement: Supplementary file 4 — Additional file 4: Fig.S3. Informative single-nucleotide polymorphisms (SNPs) flanking the breakpoint in chromosome 2 from pedigree 1 (Family LJ) and embryos based on nanopore sequencing. SNP haplotypes were identified successfully for chromosome 2, and three balanced diploid embryos (C, D, and E) were free of translocation. [file 12864_2022_9103_MOESM4_ESM.pdf]

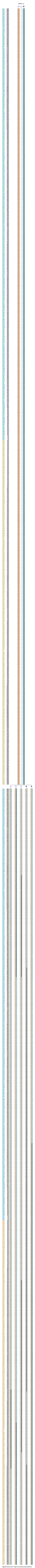

Fig. S3 Informative single-nucleotide polymorphisms (SNPs) flanking the breakpoint in chromosome 2 from pedigree 1 (Family LJ) and embryos based on nanopore sequencing. SNP haplotypes were identified successfully for chromosome 2, and three balanced diploid embryos (C, D, and E) were free of translocation.
