# Supplementary material for: Nanopore sequencing for detecting reciprocal translocation carrier status in preimplantation genetic testing
Source: BMC Genomics. 2023 Jan 2;24:1. doi: 10.1186/s12864-022-09103-5 (PMC9809107; doi:10.1186/s12864-022-09103-5)
Supplement: Supplementary file 6 — Additional file 6: Fig.S5. Informativesingle-nucleotide polymorphisms (SNPs) flanking the breakpoint in chromosome 13 from pedigree 2 (Family LHY) and embryos based on nanopore sequencing. SNP haplotypes were identified successfully for chromosome 13, and two balanced diploid embryos (A and L) were free of translocation. [file 12864_2022_9103_MOESM6_ESM.pdf]

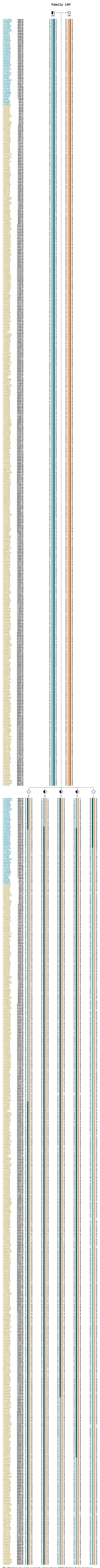

Fig. S5 Informative single-nucleotide polymorphisms (SNPs) flanking the breakpoint in chromosome 13 from pedigree 2 (Family LHY) and embryos based on nanopore sequencing. SNP haplotypes were identified successfully for chromosome 13, and two balanced diploid embryos (A and L) were free of translocation.
